# The Role of Transoesophageal Echocardiography Before Cardioversion of Atrial Fibrillation: Avoiding Overuse

**DOI:** 10.3390/life16101612

**Published:** 2026-09-26

**Authors:** Roberto Cemin

**Affiliations:** Department of Cardiology, San Maurizio Regional Hospital of Bolzano, via L. Boehler 5, 39100 Bolzano, Italy; roberto.cemin@sabes.it; Tel.: +39-0471-909950

**Keywords:** atrial fibrillation, electrical cardioversion, transoesophageal echocardiography, left atrial appendage, thromboembolism, direct oral anticoagulants

## Abstract

Atrial fibrillation (AF) is the most common cardiac arrhythmia and remains a major cause of stroke, heart failure, hospitalization, and cardiovascular mortality. Electrical cardioversion is an effective strategy for restoring sinus rhythm in symptomatic patients; however, it carries a risk of thromboembolic complications when left atrial thrombi are present. Adequate oral anticoagulation substantially reduces this risk and has become a cornerstone of peri-cardioversion management. In this setting, transoesophageal echocardiography (TOE) represents the reference imaging modality for excluding left atrial appendage (LAA) thrombus and can facilitate early cardioversion in appropriately selected patients. However, the use of TOE varies considerably across institutions and patient populations, and its role has evolved from a potentially routine examination to a more targeted diagnostic strategy. Its main value lies in patients in whom adequate and uninterrupted anticoagulation cannot be reliably established, in those with additional factors associated with an increased likelihood of thromboembolism, or when early cardioversion is clinically desirable. Conversely, routine TOE before cardioversion in patients with well-documented, adequate, and uninterrupted anticoagulation provides limited additional benefit and may not be justified given the semi-invasive nature of the examination and the procedural resources required. This review examines the pathophysiological basis of thromboembolism associated with cardioversion, current guideline recommendations, the diagnostic performance of TOE, and its evolving role in the era of direct oral anticoagulants (DOACs), with particular emphasis on the appropriate selection of patients who may benefit from a TOE-guided cardioversion strategy.

## 1. Introduction

Atrial fibrillation (AF) affects more than 37 million individuals worldwide, and its prevalence is expected to increase substantially as populations age and cardiovascular comorbidities become more common [1,2]. Beyond its well-established association with stroke and systemic embolism, AF contributes to progressive atrial remodelling, impaired cardiac function, reduced quality of life, and increased healthcare burden.

Restoration of sinus rhythm through electrical cardioversion remains an important therapeutic strategy in patients with persistent AF, particularly in those who remain symptomatic despite adequate rate control. The main safety concern associated with cardioversion is the risk of thromboembolism, which may occur because of pre-existing atrial thrombus or thrombus formation during the post-cardioversion period [3]. During AF, reduced atrial contractility promotes blood stasis, particularly within the left atrial appendage (LAA), creating favourable conditions for thrombus formation [4,5,6]. Restoration of coordinated atrial contraction may subsequently dislodge pre-existing thrombi, resulting in stroke or systemic embolism. This risk is not limited to electrical cardioversion and may also occur following pharmacological or spontaneous conversion to sinus rhythm. Moreover, transient atrial mechanical dysfunction, known as atrial stunning, persists after successful cardioversion and may contribute to continued thromboembolic risk despite restoration of sinus rhythm [7].

Historically, this risk was managed by postponing cardioversion until at least three weeks of therapeutic anticoagulation had been completed. The introduction of TOE fundamentally changed this approach by allowing direct visualization of LAA and reliable exclusion of intracardiac thrombus immediately before cardioversion. This is particularly important because the LAA is poorly visualized by transthoracic echocardiography (TTE) and represents the most common site of thrombus formation in patients with AF. The landmark Assessment of Cardioversion Using Transoesophageal Echocardiography (ACUTE) trial compared a conventional strategy of at least three weeks of anticoagulation with a TOE-guided strategy allowing early cardioversion in the absence of thrombus [8]. The study showed comparable rates of stroke, systemic embolism, and death between the two strategies, while significantly reducing the time to cardioversion in the TOE-guided group. The TOE strategy was also associated with fewer bleeding complications, largely because of shorter exposure to pre-procedural anticoagulation. These findings established TOE as a safe alternative to delayed cardioversion and provided an important foundation for subsequent guideline recommendations [1,2,8].

The introduction of direct oral anticoagulants (DOACs) further transformed peri-cardioversion management. Randomized trials on DOACs have demonstrated efficacy and safety comparable to vitamin K antagonists (VKAs), while offering more predictable anticoagulant effects and eliminating the need for routine coagulation monitoring [2,9,10,11,12]. Nevertheless, the absence of a readily available measure of treatment exposure means that uncertainty regarding adherence or treatment interruption may remain, particularly in patients with an unclear anticoagulation history. Consequently, TOE continues to play an important role in selected patients, particularly when adherence to anticoagulation is uncertain, treatment interruption cannot be excluded. thromboembolic risk is elevated, or early cardioversion is clinically desirable.

## 2. Search Strategy/Methods

This narrative review was informed by a structured literature search of the published evidence concerning TOE, LAA thrombus, anticoagulation, and cardioversion in AF. The principal databases searched were PubMed/MEDLINE, Embase, and the Cochrane Library, supplemented by manual screening of reference lists of relevant systematic reviews, randomized trials, observational studies, and contemporary international guidelines.

Search terms combined controlled vocabulary (including MeSH terms where applicable) with free-text terms relating to AF, cardioversion, TOE, LAA thrombus, anticoagulation, and direct oral anticoagulants. Representative PubMed terms included: (“Atrial Fibrillation” [MeSH] OR atrial fibrillation OR AF), AND (“Echocardiography, Transesophageal” [MeSH] OR transesophageal echocardiography OR transoesophageal echocardiography OR TEE OR TOE), AND (“Atrial Appendage” [MeSH] OR left atrial appendage OR LAA), AND (thrombus OR thrombosis), with additional combinations involving cardioversion, anticoagulation, direct oral anticoagulants, DOACs, vitamin K antagonists, and warfarin.

The search covered publications from database inception through August 2026, with particular emphasis on contemporary evidence from the era of direct oral anticoagulants. Eligible evidence included randomized controlled trials, prospective and retrospective observational studies, systematic reviews and meta-analyses, and international clinical practice guidelines addressing LAA thrombus, TOE-guided cardioversion, or peri-cardioversion anticoagulation. Studies were considered relevant when they provided data on thrombus prevalence, predictors of thrombus, diagnostic performance of TOE, clinical outcomes associated with TOE-guided cardioversion, or recommendations concerning the selection of patients for pre-cardioversion imaging.

Studies were excluded when they did not address AF or atrial flutter, did not provide clinically relevant information regarding LAA/left atrial thrombus or cardioversion, were limited to non-human research, or consisted solely of editorials, correspondence, or duplicate reports. Because this was a narrative review rather than a formal systematic review, no quantitative meta-analysis was undertaken. The evidence was therefore interpreted according to study design, patient selection, anticoagulation status, imaging strategy, and potential sources of bias.

## 3. Pathophysiological Basis of Thromboembolism During Cardioversion

The development of thromboembolism in atrial fibrillation (AF) can be understood within the framework of Virchow’s triad: blood stasis, endothelial dysfunction, and hypercoagulability [5,6]. Loss of coordinated atrial contraction reduces blood flow within the left atrium, particularly in the trabeculated left atrial appendage (LAA), which accounts for more than 90% of thrombi in patients with non-valvular AF [4].

Atrial remodelling further promotes thrombus formation through fibrosis, inflammation, dilatation, and impaired LAA contractility, favouring spontaneous echo contrast and blood stasis. This prothrombotic substrate is further amplified by conditions such as heart failure, advanced age, diabetes mellitus, hypertension, chronic kidney disease, previous stroke, and rheumatic mitral stenosis.

Importantly, restoration of sinus rhythm does not immediately normalize atrial mechanical function. Atrial stunning may persist for days or weeks after cardioversion despite recovery of electrical activity, resulting in continued impairment of atrial emptying and a transient persistence of thromboembolic risk [7]. This provides the physiological rationale for continuing therapeutic anticoagulation for at least four weeks after cardioversion, irrespective of the imaging strategy or the mode of rhythm restoration [1,2].

Residual thrombotic risk may persist even during apparently adequate anticoagulation. Studies have reported LAA thrombus in approximately 2–5% of anticoagulated patients, particularly in the presence of persistent AF, left atrial enlargement, heart failure, reduced left ventricular ejection fraction, or high CHA_2_DS_2_-VASc scores [13,14]. Thus, cardioversion should be viewed not simply as restoration of rhythm, but as a transition during which an established thrombogenic atrial substrate may temporarily persist despite electrical normalization.

## 4. Guideline Recommendations

Any rhythm-control procedure carries an inherent risk of thromboembolism. Current international guidelines provide two equivalent approaches for patients with AF lasting more than 24 h or of unknown duration [1,2].

The first consists of uninterrupted therapeutic anticoagulation for at least three weeks before cardioversion, followed by a minimum of four weeks afterward [1,2]. Although this strategy was traditionally based on vitamin K antagonist therapy, it is now equally applicable to DOACs when uninterrupted adherence can be confidently documented.

The second strategy consists of TOE-guided early cardioversion [1,8]. TOE is performed immediately before the procedure to exclude LAA thrombus. If no thrombus is detected, cardioversion may proceed without waiting three weeks, provided that therapeutic anticoagulation is initiated before the procedure and continued for at least four weeks afterward.

The 2024 ESC Guidelines for the management of atrial fibrillation assign a Class I recommendation to both approaches, emphasizing that the choice should be individualized according to clinical circumstances, the urgency of cardioversion, and the certainty of anticoagulation adherence [1]. Similarly, the 2023 ACC/AHA/ACCP/HRS guideline recognizes both strategies as appropriate and highlights the importance of uninterrupted anticoagulation irrespective of the rhythm-control strategy [2].

Particular attention is warranted when adherence to anticoagulation is uncertain. Unlike VKAs, whose therapeutic effect can be monitored through serial international normalized ratio (INR) measurements, routine laboratory assessment of DOAC activity is generally unavailable. TOE may therefore be particularly valuable when missed doses, temporary treatment interruption, or an uncertain medication history cannot be confidently excluded [1,2]. Importantly, however, the absence of LAA thrombus at the time of cardioversion does not eliminate subsequent thromboembolic risk. In patients who remain inadequately anticoagulated after cardioversion, new thrombus formation may occur during the period of atrial mechanical dysfunction (atrial stunning), before recovery of LAA contractility. Thus, TOE alone cannot compensate for inadequate post-cardioversion anticoagulation, and the procedure should be reconsidered when reliable adherence cannot be ensured.

At the other end of the risk spectrum, patients with a known AF duration of less than 24 h may generally undergo immediate cardioversion without prior TOE, even in the absence of previous anticoagulation, provided that their individual thromboembolic risk is appropriately assessed, and no high-risk features are present (Figure 1). This distinction underscores that the need for pre-cardioversion imaging depends not on AF duration alone, but on the interaction between AF duration, the underlying thromboembolic substrate, and the certainty of adequate anticoagulation.

## 5. Transoesophageal Echocardiography in Cardioversion

TOE remains the reference standard for detecting left atrial appendage (LAA) thrombus because of its high spatial resolution and close proximity to the posterior cardiac structures [1,15]. In experienced hands, its sensitivity and specificity exceed 95%. In addition to thrombus detection, TOE provides a comprehensive assessment of left atrial anatomy and function, identifying features associated with thromboembolic risk, including spontaneous echo contrast, reduced LAA emptying velocity, left atrial enlargement, valvular disease, interatrial septal abnormalities, and other potential cardiac sources of embolism.

Advanced imaging techniques may further enhance diagnostic assessment: real-time 3D TOE provides additional anatomical detail in complex LAA morphology [16], while contrast-enhanced TOE may help distinguish dense spontaneous echo contrast (“sludge”) from organized mural thrombi [17].

A reduced LAA emptying velocity, generally below 20 cm/s, is associated with increased thromboembolic risk and reflects significant mechanical dysfunction of the appendage [4]. However, hemodynamic assessment may be further refined by considering additional parameters, such as LAA ejection fraction and multiphasic Doppler flow profiles, which may provide complementary information on LAA mechanical function and blood stasis [18]. Dense spontaneous echo contrast similarly indicates marked blood stasis.

When a LAA thrombus is detected, cardioversion should be postponed and anticoagulation continued or optimized until repeat imaging confirms thrombus resolution, which may require several weeks or months [1,2].

Despite its diagnostic accuracy, TOE is a semi-invasive procedure requiring specialized equipment, trained personnel, and often conscious sedation. Complications are uncommon but include hypoxaemia, oesophageal trauma, bleeding, aspiration, arrhythmias, and, very rarely, oesophageal perforation, which has been reported in approximately 0.01–0.09% of examinations in larger series, although rates vary according to patient population and procedural setting. Contraindications should therefore be assessed systematically, including absolute contraindications such as known oesophageal perforation or significant obstruction/stricture, while conditions such as oesophageal varices, Barrett’s oesophagus, previous upper gastrointestinal surgery, recent gastrointestinal bleeding, dysphagia, cervical spine disease, coagulopathy, or thrombocytopenia are generally considered relative contraindications [19]. In patients with relative contraindications, the decision should be individualized according to the expected diagnostic benefit and procedural risk.

Routine TOE before every cardioversion is therefore not recommended. Instead, a selective approach is appropriate in patients with uncertain or inadequate anticoagulation, high thromboembolic risk, previous LAA thrombus, persistent AF of unknown duration, or a clinical need for early cardioversion [1,2,3].

Although guidelines consider uninterrupted anticoagulation and a TOE-guided strategy equivalent for elective cardioversion, TOE use varies considerably between centres and healthcare systems [1,2]. The EHRA survey of 57 European centres showed that TOE was mainly used to exclude left atrial thrombus before cardioversion, particularly when early cardioversion was required, or anticoagulation was inadequate. 71.7% of centres preferred ≥3 weeks of therapeutic anticoagulation, while 28.3% used an early TOE-guided strategy. TOE was performed in 76.1% of centres for immediate cardioversion in AF lasting >48 h or of uncertain duration, in 56.5% when anticoagulation was inadequate or uncertain, and in 47.8% of centres for patients at particularly high thromboembolic risk. Only 4.6% performed TOE routinely before every elective cardioversion [15].

These findings indicate that TOE use is influenced not only by guidelines but also by individual thromboembolic risk assessment, physician experience, and institutional practice. Previous stroke, heart failure, left atrial enlargement, long-standing persistent AF, and uncertainty regarding anticoagulation adherence may prompt TOE even when anticoagulation appears adequate [13,14,15,20,21,22].

The introduction of DOACs has simplified anticoagulation management before cardioversion because of their rapid effect, predictable pharmacokinetics, and lack of routine coagulation monitoring [11,12,23]. Trials including X-VeRT, ENSURE-AF, and EMANATE have shown very low thromboembolic event rates and support both TOE-guided and conventional cardioversion strategies [11,12,23]. However, unlike vitamin K antagonists, DOACs lack a routinely available laboratory measure capable of reliably confirming long-term treatment exposure. TOE therefore remains particularly valuable when adherence is uncertain, or treatment interruption cannot be excluded [1,2,24], but is usually performed more frequently in patients receiving DOACs than in those treated with vitamin K antagonists (39.6% versus 25.8%; *p* < 0.001), with vitamin K antagonist therapy independently associated with a lower probability of TOE (OR 0.47; 95% CI 0.33–0.67) [25].

Overall, the role of TOE has evolved from a potentially routine examination to a targeted strategy for patients in whom LAA thrombus cannot be confidently excluded or uninterrupted anticoagulation cannot be reliably established. Its use should be guided by an individualized assessment of AF duration, anticoagulation status, thromboembolic risk, and the need for early cardioversion [3,9,10,20,25].

## 6. Left Atrial Appendage Thrombus Despite Adequate Anticoagulation

Left atrial appendage thrombus (LAAT) may persist despite apparently adequate and uninterrupted anticoagulation. Observational studies and meta-analyses report a prevalence of approximately 2–7% in patients receiving oral anticoagulation before cardioversion or catheter ablation [3,13,14,21,22]. However, these estimates should be interpreted cautiously, as TOE-based cohorts may not represent the broader population of anticoagulated patients with atrial fibrillation (AF).

The meta-analysis by Lurie et al. [13], including 35 observational studies and 14,653 patients undergoing TOE after at least three weeks of oral anticoagulation, reported a pooled LAAT prevalence of 2.73% (95% CI 1.95–3.80%), with substantial heterogeneity (I^2^ = 91%). Prevalence was higher in cardioversion than ablation cohorts (5.55% vs. 1.65%), in non-paroxysmal than paroxysmal AF (4.81% vs. 1.03%), and in patients with CHA_2_DS_2_-VASc scores ≥ 3 than in those with scores ≤ 2 (6.31% vs. 1.06%).

These differences highlight the influence of patient selection and baseline thromboembolic risk. Patients referred for TOE may have persistent AF, previous thromboembolism, heart failure, left atrial enlargement, impaired ventricular function, or uncertain anticoagulation adherence. Such referral bias, together with confounding by indication, may enrich TOE cohorts for higher-risk patients and limit the generalizability of their findings.

The study by Bertaglia et al. [21] illustrates this limitation. Among 414 patients receiving at least three weeks of DOAC therapy and referred for ablation or electrical cardioversion, TOE detected LAAT in 15 (3.6%). Nearly all patients with thrombus had persistent AF. Higher CHA_2_DS_2_-VASc scores, rather than the specific DOAC used, were associated with thrombus detection. These findings confirm residual thrombotic risk but do not establish its prevalence among all adequately anticoagulated patients.

The definition of adequate anticoagulation represents another important limitation. Observational studies may rely on prescribed treatment or reported duration, although adherence and interruptions, particularly with DOACs, may be difficult to establish retrospectively. Therapeutic INR measurements provide a more readily documented indicator of anticoagulant exposure in patients receiving vitamin K antagonists (VKAs), but do not necessarily confirm uninterrupted therapeutic anticoagulation throughout the treatment period.

The indication for TOE may also contribute to heterogeneity. The higher prevalence in cardioversion than ablation cohorts reported by Lurie et al. [13] may reflect differences in AF duration, baseline risk, and anticoagulation uncertainty. Similarly, Ruzieh et al. [14] reported LAAT in 11.3% of 795 patients undergoing TOE before cardioversion. This retrospective, TOE-selected cohort showed a strong association between thrombus and advanced cardiac disease, particularly reduced left ventricular ejection fraction (LVEF). The higher prevalence likely reflects differences in cohort composition and baseline risk rather than contradictory evidence.

Overall, the frequently cited prevalence of 2–7% should be regarded as a description of selected observational TOE populations, rather than a universal estimate of residual LAAT risk in adequately anticoagulated patients with AF. Its true prevalence remains uncertain because of referral bias, confounding, heterogeneous definitions of adequate anticoagulation, and differences in patient selection.

## 7. Clinical and Echocardiographic Predictors of Persistent LAAT

The persistence of LAAT despite anticoagulation may reflect an adverse thrombogenic substrate, particularly in patients with advanced atrial cardiomyopathy and impaired atrial mechanical function (Figure 2). Among the different predictors, impaired LAA mechanical function appears to be one of the strongest determinants of thrombus persistence [3,13,14,21,22].

Clinical risk factors include heart failure, particularly with LVEF < 40%, persistent or long-standing persistent AF, previous stroke or transient ischaemic attack (TIA), advanced age, diabetes, chronic kidney disease, obesity, and high CHA_2_DS_2_-VASc scores. Several laboratory parameters have also been associated with thrombus persistence, while the coexistence of multiple risk factors may further increase its likelihood.

Echocardiographic markers provide additional information on the underlying thrombogenic substrate. An LAA emptying velocity <20 cm/s and dense spontaneous echo contrast indicate blood stasis and impaired appendage function. Severe left atrial enlargement, advanced atrial remodelling, impaired atrial mechanical function, reduced LV systolic function, and moderate or severe rheumatic mitral stenosis are also associated with increased thrombotic risk. Table 1 summarises the clinical, echocardiographic, and laboratory factors associated with persistent LAAT.

These findings help explain why selective TOE may remain valuable even in patients receiving uninterrupted DOAC therapy. Thrombus detection may identify patients with an adverse atrial substrate and impaired mechanical function, rather than simply indicating inadequate anticoagulation. Accordingly, TOE may be particularly appropriate in patients with multiple clinical, echocardiographic, or laboratory risk factors, rather than being performed routinely in every adequately anticoagulated patient [14,21,22,26].

## 8. Practical Clinical Decision-Making

Current evidence does not support routine TOE before every cardioversion. Instead, its use should be guided by an individualized assessment of thromboembolic risk and the certainty of adequate, uninterrupted anticoagulation [1,2,15,20,25]. TOE is particularly valuable in patients with AF of uncertain duration, interrupted or uncertain anticoagulation, suspected poor adherence to DOAC therapy, previous LAA thrombus or thromboembolism, persistent spontaneous echo contrast, severe left atrial enlargement, markedly reduced left ventricular ejection fraction, or multiple thromboembolic risk factors [3,14,20,21,22,23,24].

Emerging clinical prediction models, such as the CLOTS-AF score, may further support pre-test risk assessment by combining clinical and echocardiographic variables to identify patients at increased risk of LAA thrombus. Although promising, these tools require further external validation before widespread clinical implementation [20].

Conversely, patients with documented uninterrupted DOAC therapy, reliable adherence, and no major additional thromboembolic risk factors may generally undergo cardioversion without routine TOE, in accordance with current ESC and ACC/AHA/HRS recommendations [1,2]. Similarly, in patients with a known AF duration of less than 24 h and no clinical features indicating high thromboembolic risk, immediate cardioversion without prior TOE may be considered, even in the absence of chronic anticoagulation.

For appropriately selected patients with recent-onset AF, a “wait-and-see” strategy offers an additional alternative. By allowing time for spontaneous conversion, this approach may avoid unnecessary electrical cardioversion and pre-procedural imaging. The RACE 7 ACWAS trial demonstrated that delayed cardioversion following an initial wait-and-see approach was non-inferior to early cardioversion in patients with recent-onset, uncomplicated AF, with most patients converting spontaneously within 48 h [26].

These strategies reflect the relatively low thromboembolic risk associated with very recent-onset AF in appropriately selected patients. However, AF duration alone does not determine this risk, which may be substantially increased by an adverse thromboembolic substrate. Accordingly, a risk-based approach allows TOE to be reserved for patients in whom the likelihood of LAA thrombus justifies additional imaging, while avoiding unnecessary invasive procedures in those with a low residual thromboembolic risk (Figure 1).

When LAA thrombus is identified, cardioversion should be deferred and therapeutic anticoagulation continued or optimized. Management should include verification of anticoagulant adherence and dosing, as well as assessment of potentially reversible thrombotic risk factors. Repeat TOE, typically after 3–6 weeks of anticoagulation, may be performed to document thrombus resolution before reconsidering cardioversion. If thrombus persists, anticoagulation should be reassessed and optimized, followed by repeat imaging after a further treatment interval. Cardioversion should only proceed once thrombus resolution has been documented [15].

From a healthcare perspective, routine TOE before every elective cardioversion may represent an inefficient use of resources, particularly given the low prevalence of LAA thrombus among adequately anticoagulated patients. Selective imaging based on individual thromboembolic risk may therefore improve resource utilization while maintaining procedural safety. Available healthcare analyses support individualized imaging strategies, suggesting that unnecessary examinations can be reduced without increasing thromboembolic events. Furthermore, the widespread adoption of DOACs has simplified peri-cardioversion management by reducing delays associated with unstable INR values and the need for repeated monitoring with vitamin K antagonists.

Future economic evaluations should compare selective TOE with alternative strategies, including CCT-guided approaches and digital monitoring of anticoagulant adherence, to identify the most efficient and clinically appropriate approach across different healthcare systems.

## 9. Future Perspectives

The role of imaging before cardioversion is likely to evolve substantially with advances in artificial intelligence (AI), digital health, and multimodality cardiac imaging.

AI-assisted image analysis may improve automated LAA segmentation and thrombus detection on TOE and cardiac computed tomography (CCT). In addition, radiomic approaches may help distinguish true thrombus from contrast-mixing artefacts. Although promising, these applications remain largely investigational and require prospective clinical validation [27,28].

Beyond image interpretation, AI-based clinical prediction models may complement CHA_2_DS_2_-VA and conventional echocardiographic parameters by integrating clinical, laboratory, echocardiographic, and electronic health record data to estimate the individual probability of LAA thrombus. Notably, the LAT-AI model has demonstrated incremental predictive value over CHA_2_DS_2_-VASc and left ventricular ejection fraction (LVEF) in an external cohort, supporting the potential role of AI in refining established risk assessment [27]. Such approaches could facilitate a more individualized selection of patients for TOE or CCT, helping to identify those in whom imaging might be safely avoided and those who may benefit from imaging despite apparently adequate anticoagulation.

However, promising predictive performance alone is insufficient to support routine clinical implementation. AI-based models require robust external validation in independent, contemporary, and clinically diverse populations, with careful assessment of discrimination and clinical utility. Prospective impact studies should subsequently establish whether model-guided imaging strategies improve patient selection without increasing thromboembolic or bleeding risk. Until such evidence becomes available, AI-based prediction should remain complementary to established clinical and echocardiographic assessment rather than replace it.

Digital health and remote monitoring technologies may further support individualized decision-making by improving the assessment of DOAC adherence. More reliable documentation of uninterrupted anticoagulation could increase confidence in selecting patients for cardioversion without prior imaging, provided that established clinical and anticoagulation requirements are met.

Advances in cardiac imaging may also broaden the range of options available for excluding LAA thrombus. CCT, particularly with delayed contrast acquisition, is emerging as a promising alternative to TOE, offering high diagnostic accuracy, with reported sensitivities above 95% and negative predictive values approaching 100% [10,29]. Nevertheless, its use remains constrained by radiation exposure, the need for iodinated contrast, and its inability to assess functional parameters such as spontaneous echo contrast and LAA emptying velocity. Cardiac magnetic resonance (CMR) may provide complementary information on atrial fibrosis and structural remodelling, although its role in pre-cardioversion thrombus assessment requires further investigation.

Future research should therefore focus on prospective studies evaluating integrated strategies combining clinical risk assessment, selective TOE, CCT, CMR, and digital adherence monitoring. The ultimate goal is to determine which patients derive the greatest benefit from imaging and to develop a more personalized, efficient, and less invasive approach to thromboembolic risk assessment before cardioversion.

## 10. Conclusions

Current evidence supports a personalized, risk-based approach to imaging before cardioversion. Routine TOE is not required in every patient with AF, particularly when uninterrupted anticoagulation is well documented and the overall thromboembolic risk is low. Conversely, imaging remains particularly relevant when the adequacy of anticoagulation is uncertain, when clinical or echocardiographic features suggest a higher likelihood of LAA thrombus, or when early cardioversion is clinically advisable before completion of the recommended 3-week period of adequate anticoagulation.

More than two decades after the ACUTE trial, TOE remains the reference imaging modality for excluding LAA thrombus and enabling safe early restoration of sinus rhythm. The widespread use of DOACs has simplified peri-cardioversion anticoagulation and substantially reduced thromboembolic risk, but the optimal use of TOE in anticoagulated patients remains an area of clinical debate.

Overall, current evidence from clinical trials, registries, and international guidelines favours a selective rather than universal TOE strategy. Patients with reliable anticoagulation can generally undergo cardioversion without routine imaging, while TOE may provide additional diagnostic value in selected high-risk patients. Future approaches, including cardiac computed tomography, artificial intelligence-based risk prediction, and digital adherence monitoring, may further improve patient selection. Until these strategies are adequately validated, TOE will remain an important tool for individualized thromboembolic risk assessment before cardioversion.

## Figures and Tables

**Figure 1:**
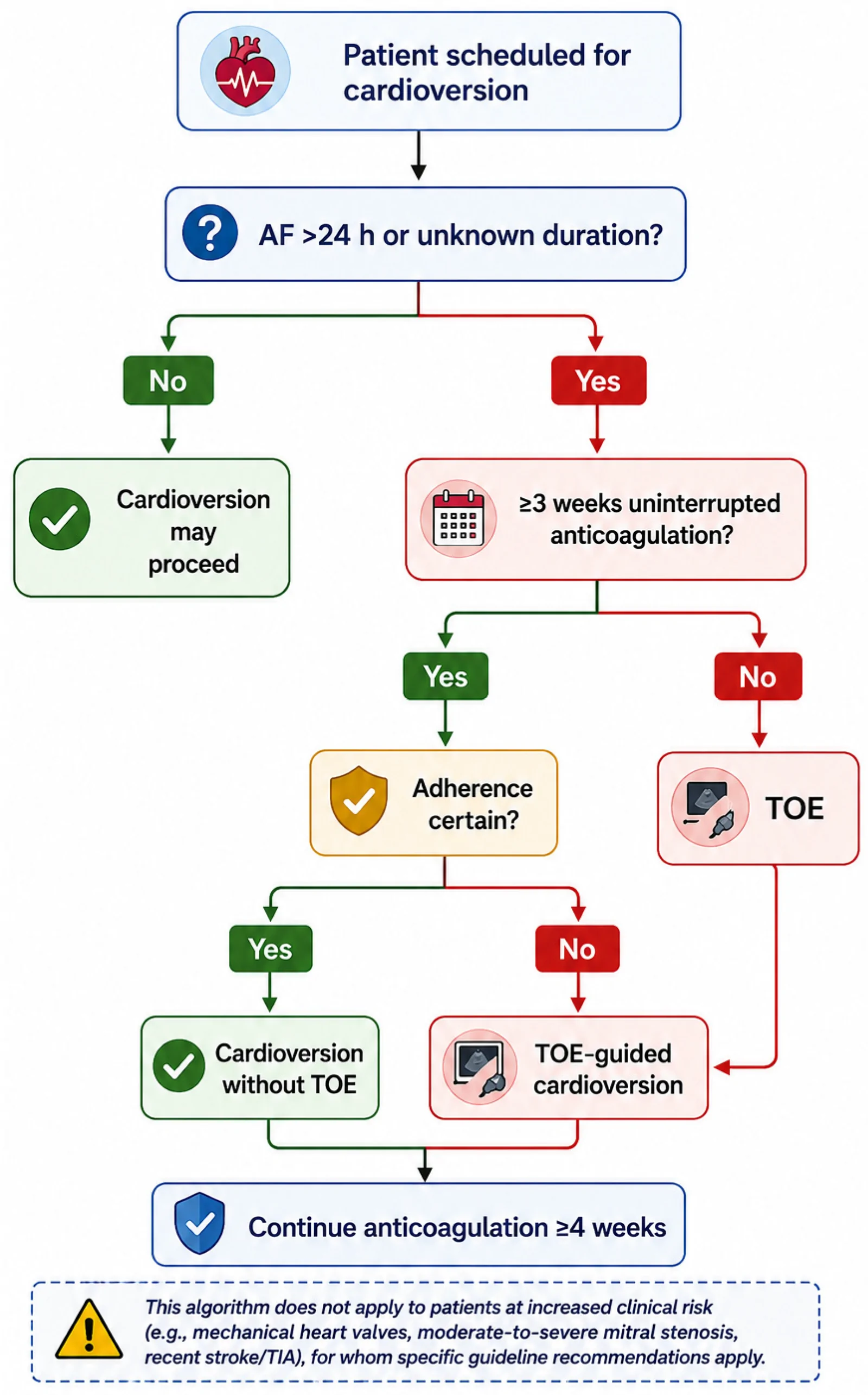
Suggested clinical algorithm for TOE before cardioversion.

**Figure 2:**
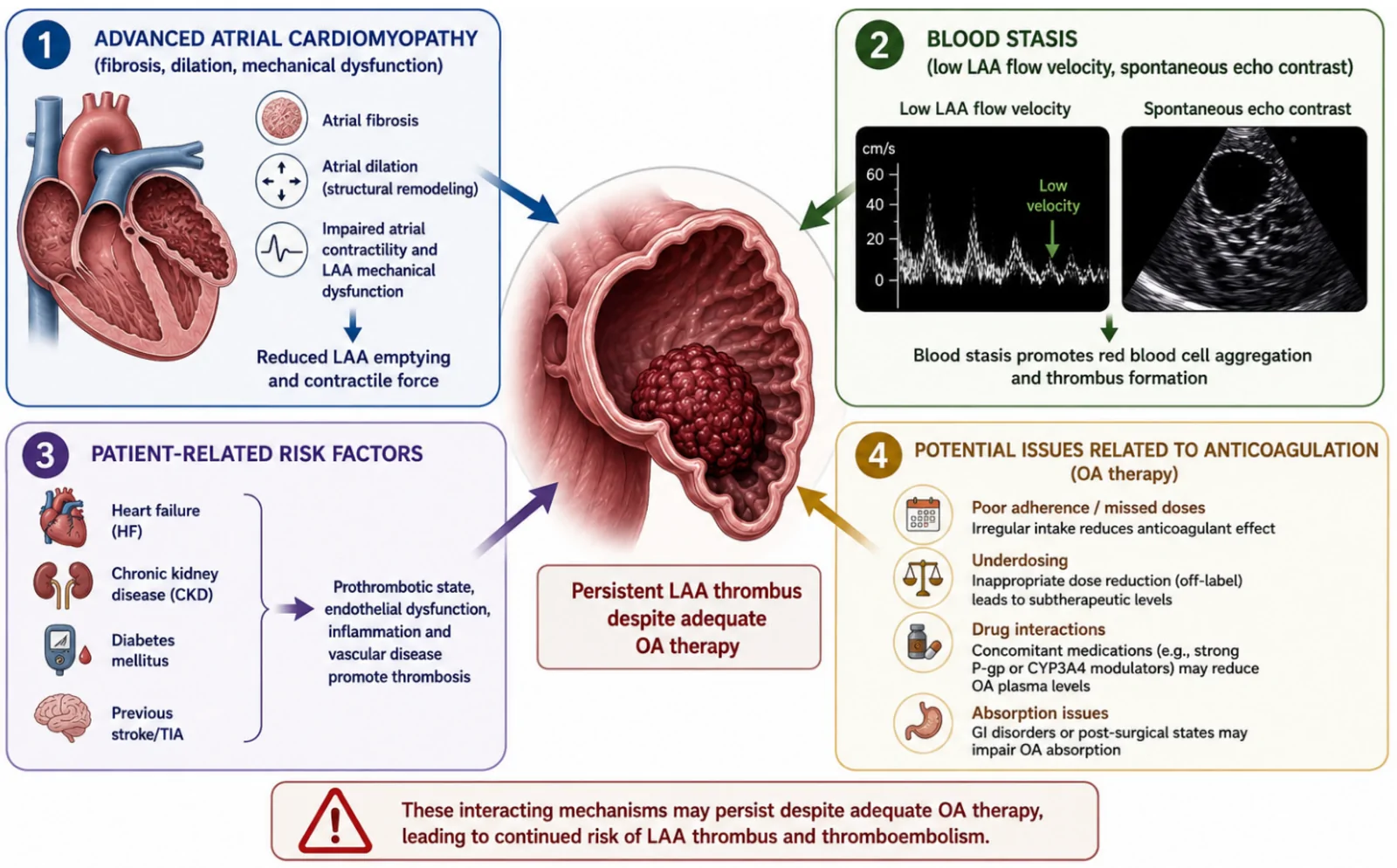
Mechanisms responsible for persistent left atrial appendage (LAA) thrombus despite adequate oral anticoagulation (OA) therapy.

**Table 1:** Predictors of persistent left atrial appendage thrombus despite OAC therapy.

Domain	Predictors
**Clinical**	**Persistent/long-standing persistent AF**, previous stroke/TIA, heart failure, LVEF < 40%, CHA_2_DS_2_-VASc ≥ 3, diabetes mellitus, chronic kidney disease, obesity
**Echocardiographic**	**LAA emptying velocity < 20 cm/s**, dense spontaneous echo contrast, severe left atrial enlargement, low LAA ejection fraction, complex LAA morphology, advanced atrial remodelling, **moderate or severe rheumatic mitral stenosis**
**Laboratory/Other**	**Elevated NT-proBNP**, elevated D-dimer, renal dysfunction, elevated inflammatory biomarkers

**Abbreviations:** AF, atrial fibrillation; CHA_2_DS_2_-VASc, congestive heart failure, hypertension, age ≥75 years, diabetes mellitus, previous stroke/TIA/thromboembolism, vascular disease, age 65–74 years, sex category; OAC, oral anticoagulant; LAA, left atrial appendage; LVEF, left ventricular ejection fraction; NT-proBNP, N-terminal pro-B-type natriuretic peptide; TIA, transient ischaemic attack; **in bold the strongest predictors**.

## Data Availability

No new data were created or analyzed in this study. Data sharing is not applicable to this article.
